# Mycoplasma Pneumonia and Influenza B Co-infection in an Adult

**DOI:** 10.7759/cureus.113820

**Published:** 2026-08-02

**Authors:** Hardik Mehta, Atefeh Kalantary, Sina Shafiei

**Affiliations:** 1 Internal Medicine, University of California Los Angeles, Los Angeles, USA

**Keywords:** community acquired pneumonia (cap), hospitalization, influenza, influenza virus type a, mycoplasma pneumonia

## Abstract

Community-acquired pneumonia (CAP) is a significant cause of morbidity and mortality among adults worldwide, frequently resulting in prolonged hospitalization. Co-infection with both viral and bacterial microorganisms is an important contributor to this morbidity. *Streptococcus pneumoniae* is the most common causative agent of CAP; however, including atypical bacteria in the differential diagnosis may facilitate timely treatment and improve patients' outcomes. Here, we present a 50-year-old female with a recent household exposure to influenza B who developed progressive hypoxemia despite outpatient management and required hospitalization. In-hospital testing confirmed *Mycoplasma pneumoniae* infection; influenza A/B testing at admission was negative, consistent with resolution of her preceding, non-laboratory-confirmed influenza B illness. Appropriate treatment resulted in resolution of hypoxemia and successful discharge. This case underscores the importance of early consideration of atypical bacterial co-infections in patients presenting with severe CAP, which may reduce the likelihood of prolonged hospitalization.

## Introduction

Community-acquired pneumonia (CAP) is one of the leading causes of infection-related morbidity and mortality worldwide, accounting for millions of hospitalizations annually and carrying a 30-day mortality rate of 5-15% in patients requiring inpatient care [1,2]. The clinical spectrum of CAP ranges from mild illness managed in the outpatient setting to severe disease necessitating intensive care unit admission. Accurate identification of the causative pathogen remains central to guiding appropriate antimicrobial therapy and improving patient outcomes.

Respiratory viral infections are well-recognized predisposing factors for secondary bacterial pneumonia. Influenza, in particular, disrupts the integrity of the airway epithelium and impairs innate immune defenses, creating a permissive environment for bacterial superinfection [3]. While *Streptococcus pneumoniae* and *Staphylococcus aureus* are the most frequently implicated bacterial co-pathogens in post-influenza pneumonia, atypical organisms such as *Mycoplasma pneumoniae* are considerably less common, comprising approximately 6% of bacterial co-infections in CAP [4,5]. *Mycoplasma pneumoniae* is an obligate intracellular pathogen characterized by a prolonged incubation period of two to three weeks, a predominantly atypical clinical presentation, and the absence of a cell wall, rendering it intrinsically resistant to beta-lactam antibiotics [6].

Co-infection with influenza and *Mycoplasma pneumoniae* has been reported in the literature but remains rare, and its occurrence in adults is infrequently described [7]. Given the diagnostic challenge posed by overlapping symptoms, delayed serologic confirmation, and the potential for rapid clinical deterioration, heightened clinical suspicion is essential. Here, we present a case of *Mycoplasma pneumoniae* and influenza B co-infection in a previously healthy adult who developed hypoxemic respiratory failure requiring hospitalization, highlighting the importance of including atypical pathogens in the differential diagnosis of severe CAP following influenza illness.

## Case presentation

A 50-year-old female with no past medical history presented to her primary care physician's office due to hypoxia. Approximately two weeks prior, her daughter had received a confirmed diagnosis of influenza B; the patient herself was not tested for influenza at that time, and her presumed influenza B illness was diagnosed on clinical and exposure grounds alone, rather than by laboratory confirmation. She subsequently developed fevers, which had since resolved, followed by a productive cough with yellow-green sputum. She had not received an influenza vaccination earlier in that season, and her vaccination history was otherwise not documented (e.g., pneumococcal and COVID-19 vaccination status were unknown). She had no smoking history. Her family history was not significant for interstitial lung disease. She worked as a kindergarten teacher. She denied shortness of breath, chest pain, back pain, and dizziness but reported increased fatigue over the preceding week. She returned to her primary care physician's office, where an oxygen saturation of 87-92% on room air was identified during a routine vitals check.

In the emergency department, her oxygen saturation was 89-90% on room air, improving to greater than 92% with 2 L of supplemental oxygen via nasal cannula. Respiratory rate was 12-16 breaths per minute, and other vital signs were within normal limits; no arterial blood gas was obtained, and oxygenation was assessed by pulse oximetry throughout the admission. Using these values, her calculated CURB-65 score was 0, and her estimated Pneumonia Severity Index (PSI) was Class I-II, both of which would traditionally suggest low mortality risk; her hospitalization was instead driven by refractory hypoxemia not captured by either composite score. On physical examination, coarse crackles were auscultated, more prominent over the right lung than the left. Relevant laboratory findings are summarized in Table 1.

**Table 1:** Laboratory findings

Variable	On Presentation	Reference Ranges
White cell count (per μL)	10,200	4000–11,000
Differential count (%)		
Neutrophils	76.2	40.0–70.0
Lymphocytes	15.0	20.0–40.0
Monocytes	6.6	0.0–11.0
Eosinophils	1.3	0.0–8.0
Basophils	0.9	0.0–2.0
Hematocrit (%)	35.4	37.0–47.0
Hemoglobin (mg/dL)	12.1	11.7–15.7
Platelet count (per μL)	494,000	150,000–400,000
Sodium (mmol/L)	137	136–145
Potassium (mmol/L)	3.8	3.6–5.1
Chloride (mmol/L)	105	98–107
Carbon dioxide (mmol/L)	27	21–32
Urea nitrogen (mg/dL)	9	7–18
Creatinine (mg/dL)	0.82	0.55–1.02
Glucose (mg/dL)	120	74–99
C-reactive protein (mg/L)	20.4	<10
Erythrocyte sedimentation rate (mm/hr)	90	0–30
*Mycoplasma pneumoniae* IgG (U/mL)	1235	0–99
*Mycoplasma pneumoniae* IgM (U/mL)	6237	0–769
ANA speckled pattern	1:160	< 1:80
Sputum culture	Negative	Negative
Blood culture	Negative	Negative
Urine *Legionella* antigen	Negative	Negative
Beta-D-glucan	Negative	Negative
Coccidioidomycosis serology	Negative	Negative

**Figure 1:**
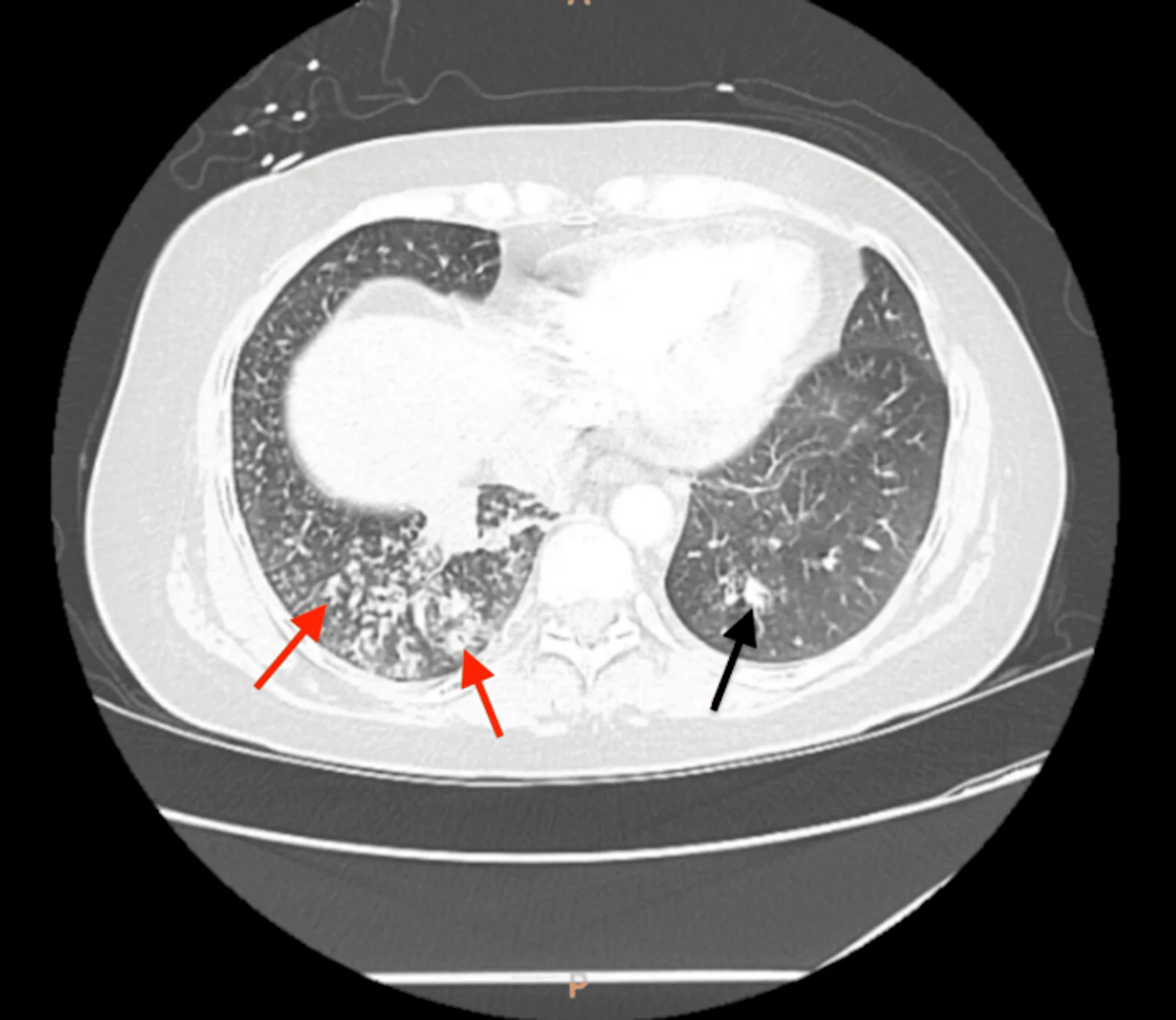
Contrast-enhanced CT of the chest. An axial image of the chest shows scattered ground-glass opacities with tree-in-bud nodularity predominantly at the right lower lobe (red arrows); however, they are also present at the left lower lobe (black arrow).

The complete blood count was within normal limits with a mild left shift. Erythrocyte sedimentation rate and C-reactive protein were elevated. Procalcitonin testing was not available at the institution. COVID-19 and influenza A/B nasal swab testing returned negative, consistent with resolution of her preceding, unconfirmed influenza B illness. Chest X-ray demonstrated mild interstitial infiltrates at the lung bases. Given her persistent, unexplained hypoxia, a CT pulmonary angiogram with PE protocol was obtained and did not demonstrate pulmonary embolism; however, diffuse ground-glass opacities with tree-in-bud nodularity were identified, predominating in the right lower lobe (Figure 1).

Empiric intravenous antibiotic therapy was initiated with ceftriaxone and azithromycin. Given her persistent hypoxia and lack of improvement despite outpatient management, together with the atypical CT findings described above, an extended workup was pursued to evaluate for both autoimmune and less common infectious etiologies beyond typical bacterial CAP. Bacterial and AFB sputum cultures, blood cultures, urine *Legionella* antigen, beta-D-glucan, and coccidioidomycosis serology were obtained (results summarized in Table 1). An extended autoimmune serologic workup was also sent, including ANA (antinuclear antibodies), anti-dsDNA, SSA (Sjögren's syndrome-related antigen A), SSB (Sjögren's syndrome-related antigen B), rheumatoid factor, and complement C3/C4. Pulmonology was consulted and offered bronchoscopy; however, after a thorough discussion of the risks and benefits, the patient elected to defer this diagnostic procedure.

The patient's oxygen requirements worsened on hospital day 3, necessitating escalation to high-flow nasal cannula (HFNC). Pulmonology initiated systemic corticosteroid therapy with prednisone 60 mg daily. Over the next several days, the patient's oxygen requirements improved, and she was weaned to 2 L via nasal cannula. Serologic results returned within normal limits, with the exception of elevated *Mycoplasma* IgM, *Mycoplasma* IgG, and ANA with a speckled pattern (Table 1). She completed a seven-day course of ceftriaxone and azithromycin. The patient was discharged on hospital day 10 on a tapering course of oral prednisone, and no further antimicrobial therapy was prescribed upon discharge.

## Discussion

CAP is one of the main causes of death in adults worldwide. The incidence of CAP has increased after the COVID-19 pandemic. Co-infection with viral and bacterial pathogens has been reported in many of these cases. The most common bacterial pathogen in co-infections is *Streptococcus* (36%), whereas atypical pathogens (for example, *Mycoplasma pneumoniae*) are considerably rarer, accounting for only 6% of these cases [4,5]. Research by Lim et al. in 2019, in which 235 patients with CAP were studied, suggested that one in five patients with severe CAP has concurrent bacterial and viral infection. Co-infection also demonstrated a much higher mortality rate compared to solitary viral infection. Characteristics more commonly seen in co-infections compared to viral infections alone include higher CRP levels, a higher percentage of neutrophil count, and higher temperature. The etiology of viral-bacterial co-infection is not fully understood; one contributing mechanism may be disruption of the epithelial barrier and dysfunction of the immune system due to upregulation of adhesion proteins [6]. Influenza virus can replicate in epithelial cells, leading to damage of the airway epithelium. This can lead to increased susceptibility to bacterial infections, as seen in this case. Furthermore, Sumitomo et al. suggested that extracellular platelet-activating factor receptor (PAFR) binds to numerous respiratory bacterial pathogens, which leads to worsening of bacterial burden and bacteremia. In addition, the co-infection of viral and bacterial pathogens can cause exudation of fluids, erythrocytes, and leukocytes into the alveolar space, leading to ventilation/perfusion impairment that contributes to severe respiratory insufficiency, as seen in this patient [8].

Comparisons with previously published cases of adult influenza B and *M. pneumoniae* co-infection are limited. Shinozaki et al. described a 19-year-old woman whose co-infection was confirmed by sputum PCR and serology after her pneumonia progressed despite initial anti-influenza and beta-lactam therapy; her CT demonstrated dense lobar consolidation, in contrast to the ground-glass opacities and tree-in-bud nodularity seen in our patient, and she required escalation to meropenem and levofloxacin before improving [7]. In a larger retrospective cohort of 244 adult and pediatric influenza pneumonia patients, Kim et al. found that *M. pneumoniae* co-infection was strongly associated with age 5-10 years (adjusted odds ratio 18.83, 95% CI 5.90-60.08) and was otherwise clinically indistinguishable from influenza pneumonia without the co-infection; the authors noted that the clinical utility of routine *M. pneumoniae* testing is likely lower in adults, given the lower background frequency of *M. pneumoniae* CAP in that population [9]. Our patient, at age 50, falls well outside this reported high-risk pediatric age band. This case suggests that, while population-level testing yield may be low in adults, individual adult patients with a compatible clinical and radiographic picture - persistent or worsening hypoxemia despite standard therapy and atypical infiltrates on imaging - still warrant consideration of atypical co-pathogens such as *M. pneumoniae*, regardless of age.

Therefore, it is important to start antibiotics early in these patients. A double-blind, multicenter, randomized controlled trial by Blum et al. demonstrated that adjunct corticosteroid use decreased median time to clinical stability from 4.4 to 3.0 days and median time to hospital discharge from seven to six days in patients with CAP (hazard ratio 1.19, 95% CI 1.04-1.38, P = 0.012), without an increase in complications [8]. Our patient's total hospital stay was 10 days, longer than the median discharge times reported in either study arm of Blum et al.; this likely reflects the added complexity of a co-infection requiring an extended diagnostic workup and escalation to HFNC before corticosteroids were introduced, rather than a directly comparable clinical course, and should not be interpreted as evidence of a treatment effect. In our patient, corticosteroids were initiated on hospital day 3 - after escalation to HFNC for worsening hypoxia - concurrently with an antibiotic course that had already been running for three days; she was weaned back to 2 L nasal cannula over the following days and discharged on hospital day 10 on a tapering prednisone course. Because both therapies overlapped, this case cannot isolate the relative contribution of antimicrobial therapy versus corticosteroids to her clinical improvement. This uncertainty is consistent with the broader adult literature: a recent retrospective cohort of hypoxemic adults hospitalized with *M. pneumoniae* pneumonia found that adjunctive corticosteroid treatment was not associated with a significantly shorter time to resolution of hypoxemia or length of stay, although fever duration was shorter, with no increase in complications [10,11]. We therefore present her improvement as temporally associated with, rather than proven to be caused by, corticosteroid initiation.

Co-infections of influenza and mycoplasma have been reported but continue to be rare. Given the household exposure and reported symptom onset roughly two weeks before admission, and the differing incubation periods generally described for influenza (1-3 days) and *M. pneumoniae* (2-3 weeks), the clinical timeline is compatible with near-simultaneous acquisition of both pathogens; however, because the initial influenza B illness was not laboratory-confirmed, this sequence cannot be established with certainty. Although the frequency of *M. pneumoniae* is lower than that of other bacterial pathogens, it should still be considered as one of the causative pathogens in influenza virus co-infections [9].

This case has several limitations. First, the diagnosis of *M. pneumoniae* infection relied on IgM and IgG serology rather than PCR; serologic testing can remain positive for weeks to months after a prior infection and is subject to false-positive results from cross-reactivity, whereas PCR would have provided more specific confirmation of active infection [4]. Second, the patient's initial influenza B illness was never laboratory-confirmed; her presumptive diagnosis was based on a household exposure and a compatible symptom course, which limits our ability to characterize the true timing and confirm the causative role of influenza in this presentation. Third, no follow-up imaging was obtained after this hospitalization, and no coronal reconstruction of the original CT pulmonary angiogram is available, limiting our ability to document radiographic improvement.

## Conclusions

In conclusion, patients infected with a virus and a bacterial pathogen more often develop severe CAP and have a longer hospitalization than those with a bacterial etiology alone. Although *Mycoplasma pneumoniae* accounts for a minority (approximately 6%) of bacterial co-infections in CAP, clinicians should consider atypical pathogens - including *M. pneumoniae* - in patients with persistent or worsening respiratory symptoms despite standard antimicrobial and supportive therapy, particularly when symptom onset follows a recent influenza illness by two to three weeks.
